# Unveiling the Disparities in the Field of Precision Medicine: A Perspective

**DOI:** 10.1002/hsr2.71102

**Published:** 2025-07-27

**Authors:** Maha Hosni Morsi, Bashaer Elawfi, Saad Ashraf ALsaad, Ahmed Nazar, Hamed Abdelma'aboud Mostafa, Sara Adel Awwad, Maya Magdy Abdelwahab, Husam Tarakhan, Ehssan Baghagho

**Affiliations:** ^1^ Clinical and Chemical Pathology Department, Faculty of Applied Health Sciences Technology Misr University for Sciences and Technology Giza City Egypt; ^2^ Medical Research Group of Egypt Negida Academy LLC Arlington Massachusetts USA; ^3^ Faculty of Medicine Sana'a University Sana'a Yemen; ^4^ Faculty of Applied Medical Sciences Jerash University Jerash Jordan; ^5^ College of Medicine University of Baghdad Baghdad Iraq; ^6^ Faculty of Medicine Al‐Azhar University Dameitta Egypt; ^7^ Faculty of Medicine Jordan University of Science and Technology Irbid Jordan; ^8^ Faculty of Medicine Helwan University Cairo Egypt; ^9^ Faculty of Medicine Hashemite University Zarqa Jordan; ^10^ General Organization for Teaching Hospitals and Institutes (GOTHI) Cairo Egypt

**Keywords:** disparities in health, individualized, personalized medicine, public health, theranostics

## Abstract

**Background and Aim:**

Precision medicine prescribes medication based on genetic data, social history, and environment, offering more effective care. This article presumes that many disparities shatter this promise for numerous groups. The article discusses the challenges of personalized medicine in relation to disparities, proposes solutions, and introduces strategies to address these issues.

**Methods:**

A comprehensive literature review was done to understand precision medicine, its implementation challenges, and possible answers. Academic databases, including Google Scholar, PubMed, and Scopus, were searched for relevant studies and peer‐reviewed articles. Peer‐reviewed precision medicine articles in oncology, internal medicine, public health, and obstetrics and gynecology were included to provide a broad and interdisciplinary perspective.

**Results:**

The concept of precision medicine needs to be implemented throughout many levels of society and healthcare systems around the world. Access to genomic data is limited in high‐income countries, and socioeconomic disparities hinder healthcare equality, particularly for low‐income individuals or those without insurance. The digital divide, lack of education, ethical concerns, and regulatory frameworks contribute to disparities in personalized medicine. We believe that health equity will be achieved by addressing these discrepancies and suggesting some strategies to overcome them.

**Conclusion:**

Precision medicine has successfully treated and helped in the early detection of many diseases, like severe asthma, cancer, and type 1 diabetes. Socioeconomic position, education, data, access, and regulatory frameworks prevent minorities and low‐income populations from using it. For instance, lack of awareness and other inequalities in access to precision medicine limit T1DM HLA typing and autoantibody surveillance. These discrepancies must be addressed to improve minority and low‐income T1DM patients' outcomes. The patient‐oriented strategy is cost‐effective and includes benefits through education, genetic data diversity promotion, and increased access, but regulatory frameworks are essential.

**Abbreviations**
ASMs: antiseizure medications
CDSS: computerized decision support systems
EHR: electronic health records
ERS: endoplasmic reticulum stress
FDA: Federal Drug Administration
FDCA: The Federal Food, Drug, and Cosmetic Act
GINA: The Genetic Information Nondiscrimination Act
GLP‐1: glucagon‐like‐peptide‐1
HER2/neu/EGFR: human epidermal growth factor receptor 2, neu, and epidermal growth factor receptor
NGS: next‐generation sequencing
PHSA: Public Health Service Act
PMI: precision medicine initiative
RA: receptor agonist
SLE: systemic lupus erythematosus
SLEDAI: Systemic Lupus Erythematosus Disease Activity Index
WWE: women with epilepsy

## 1 Introduction

Precision medicine involves prevention and treatment strategies that take individual variability into account [1]. Precision medicine is a medical approach that customizes medical treatments based on an individual's specifications, such as lifestyle and environment, as well as specific clinical and molecular characteristics of the illness. These may or may not include the 1% differences in DNA that distinguish individuals [2]. The precision medicine approach contrasts with the conventional “one size fits all” or “average patient” idea, which may not accurately reflect the prevailing practice in modern medicine [1, 3].

The origins of precision medicine are not precisely known [4, 5]. In the late 19th century, the renowned Canadian doctor Sir William Osler stated that “The good physician treats the disease; the great physician treats the patient who has the disease” [6, 7]. Although the idea of precision medicine existed during Sir William Osler's time, the Precision Medicine Initiative (PMI), introduced by Barack Obama in 2015, significantly boosted its prominence [2]. Modern medicine has adopted a pharmacogenomics‐enabled genotype‐focused patient care approach to improve clinical efficacy and expand medical care based on health needs. This approach aims to predict patients' responses to recommended treatments, enabling better patient care. For example, Trastuzumab, the first “personalized” drug for breast cancer, is effective in patients with amplification of HER2/neu/EGFR, which is 10%–20% of all patients [8]. Nowadays, severe asthma treatment is becoming more personalized with the introduction of antibodies specific to phenotypes or endotypes [9]. With this in mind, omalizumab, an anti‐IgE medication, was authorized for use by allergic asthma patients whose serum IgE levels are high [10]. On the other hand, patients suffering from severe eosinophilic asthma are prescribed mepolizumab, reslizumab, and benralizumab, anti‐IL‐5/Rα medications [11]. Targeted biologic therapy effectively treats severe asthma and improves personalized medicine for chronic lung diseases like obstructive pulmonary disease and idiopathic pulmonary fibrosis [12]. COVID‐19 has highlighted the necessity to discover clinical and biological risk factors as well as long‐term consequences for acute infectious diseases [13]. By deepening our knowledge of pathogenic pathways of COVID‐19 (endotypes) and accurately categorizing patient symptoms and disease patterns (phenotypes), we can identify more reliable biomarkers and diagnostic methods. This improved understanding could enable more targeted interventions, individualized drug treatments, and innovative biological therapies [7]. Another example is precision medicine in the context of type 1 diabetes. Type 1 diabetes is an autoimmune disease caused by the host immune system destroying insulin‐producing pancreatic beta cells in response to a foreign antigen in a genetically susceptible person [14]. Nearly half of the genetic risk is in genes in the human major histocompatibility complex (MHC), primarily the HLA [15]. Diagnosis is being improved through the use of standardized care practices, enhanced testing methods, and the inclusion of novel biomarkers. It is also recognized that type 1 diabetes is a heterogeneous condition, and categorizing it into subclasses within different populations can lead to better targeted treatments, improved glycemic control, and reduced complications, ultimately enhancing long‐term quality of life. For instance, by downregulating the expression of MHC class I proteins, GLP‐1 RAs may provide a means to avoid the beta cell's “unmasking” to immune effector cells. As an addition to insulin, the GLP‐1 analog liraglutide lowers HbA1c and decreases body weight in type 1 diabetes [16]. Moreover, a recent study by Wang and colleagues sheds light on the role of ERS in inducing β‐cell‐targeted autoimmunity and can be used to prevent and cure T1D. They found that endoplasmic reticulum stress (ERS) altered the MIP of β‐cells and boosted the MHC‐I presentation of conventional self‐peptides [17].

Monitoring the physiological state is a crucial component of precision medicine, which is applied in diagnostics, therapeutics, prevention, and prognostics [18]. Precision monitoring can look at biological markers (e.g., continuous glucose monitoring), behaviors (e.g., physical activity), food, sleep, and psychophysiological stress. Digital apps, cutaneous/subcutaneous sensors, ingestible sensors, blood assays, etc., enable precision monitoring. Success depends on intelligent processing, integration, and interpretation of precise monitoring data [19].

In addition to insulin being the primary treatment for type 1 diabetes, significant progress has been made in developing interventions to delay its onset. Most type 1 diabetes precision medicine prevention efforts involve immune interventions, such as beta cell preservation with T cell targets (teplizumab/anti‐CD3, abatacept/anti‐CD80 and anti‐CD86), B cell targets (rituximab/anti‐CD20), and inflammatory cytokines (anti‐TNF‐alpha, anti‐IL‐6R) [20, 21].

Pharmacogenetics is emerging as a promising approach for personalizing antiseizure medication (ASM) treatment in women with epilepsy (WWE) [22]. This approach examines how genetic variations affect drug metabolism and effectiveness. While this offers potential for more targeted treatment, it raises ethical considerations, particularly for pregnant women. Only a small number of the ~30 FDA‐approved medications for epilepsy have been examined for their malformation side effects. Thus, the genetic contributions to the risks of ASM‐induced malformations for the majority of ASMs are still unknown, and even for those ASMs that are considered to be safe for WWE during pregnancy, positive outcomes are not always guaranteed [22]. The field combines genetic insights with population‐based modeling to determine optimal drug concentrations based on individual patient characteristics, though implementing such personalized treatment remains complex [23].

Precision medicine concepts suggest a combination of therapies for specific risk subgroups [21]. Looking ahead, the application of precision medicine to type 1 diabetes holds the potential for early identification of at‐risk individuals, classification into subgroups for targeted interventions, and optimization of treatment to minimize the risk of complications in those who develop the disease [14].

Precision medicine is promising, but it is inequitably implemented, especially to minority and underrepresented ethnic populations [24]. To tackle the barriers to accessing precision medicine, it is essential to comprehend these issues and leverage understanding to create community‐focused strategies aimed at reaching the underrepresented populations [24]. This article aims to explore the limits of precision medicine and the consequences of health disparities. Several logistical, scientific, and cultural challenges have hindered the progress of precision medicine over the past years. Using the perspective of global health inequalities, this piece of work aims to define the obstacles to precision medicine and propose solutions.

## 2 Methods

A comprehensive literature review examined disparities in the implementation and access to precision medicine across healthcare systems and possible answers. Academic databases, including Google Scholar, PubMed, and Scopus, were thoroughly searched to discover relevant studies and peer‐reviewed articles that have investigated the subject matter. To connect MeSH terms and keywords during our search, we used Boolean operators, such as “AND” and “OR.” We used the following keywords: ((Medicine, Precision) OR “Precision Medicine”[Mesh] AND “Healthcare Disparities”[Mesh]).

The inclusion criteria were peer‐reviewed, English language publications with full‐text availability and a clear focus on precision medicine disparities, in the last 10 years, to provide a comprehensive and interdisciplinary viewpoint on the matter. Our search initially identified 354 potentially relevant articles. After removing 219 duplicates using Zotero reference management software, 135 unique articles remained for screening. After excluding non‐peer‐reviewed sources (such as preprints and opinion pieces), articles that focus on a single disease or condition without consideration of broader implications for precision medicine, and studies published more than 10 years ago unless they provide foundational insights relevant to current discussions and articles that were not in English [25], articles remaining. By synthesizing information from the selected papers, this perspective sought to provide a comprehensive and evidence‐based assessment.

## 3 Precision Medicine and the Existing Health Disparities

While precision medicine has significant promise to enhance individualized treatment, it may exacerbate current health inequities due to many variables. Restricted access to genomic data, socioeconomic factors, financial constraints, challenges in developing requisite infrastructure for an effective information system, gender inequalities, insufficient education and awareness, ethical dilemmas, and a shortage of regulatory frameworks constitute obstacles that hamper access to precision medicine.

### 3.1 Disparities in Genetic Testing Access

Significant discrepancies and limitations are revealed across various dimensions in the global landscape of genetic testing [26]. Geographic representation in genomic data collection is heavily skewed toward Europe [26], while testing infrastructure in some African regions is extremely concentrated; for example, just five countries—South Africa (38%), Kenya (14%), Morocco (9%), Nigeria (6%), and Egypt (5%) —make up 71% of next‐generation sequencers [27]. Research quality and population‐wide applicability are directly affected by this uneven distribution. Genetic disorder tests are still expensive because they are confined to private laboratories and are often outsourced. This is exemplified by Egypt's breast cancer screening program, which has trouble providing genetic testing owing to extremely high costs [28].

Additionally, precision medicine confronts considerable obstacles in terms of racial and ethnic representation in genetic research and clinical applications. A key worry is the underrepresentation of racial and ethnic minorities in genetic studies, which has a direct impact on treatment efficacy. For example, current carrier screening panels, while including over 1000 alleles for more than 200 illnesses, are primarily composed of alleles common to persons of European descent, limiting their utility for people of different ancestries [29]. This underrepresentation has ethical and scientific concerns, since a lack of understanding of genetic variation among populations impedes worldwide prognosis, illness prevention, and therapeutic identification [30].

### 3.2 Disparities in Precision Medicine Treatments Access

Access constraints to precision medicine resources create a complicated web of issues for healthcare delivery and results. While next‐generation sequencing (NGS) testing has shown promise, with 72% of precision medicine tests improving health outcomes, availability is still restricted, particularly in rural and underserved areas, and only 20% of these tests result in cost savings [31, 32]. The difficulties extend beyond diagnostics to treatment availability, as indicated by one‐third of patients who underwent NGS testing being unable to obtain FDA‐approved targeted drugs, while cost barriers impair prescription adherence for 34% of uninsured Americans and 11% of those aged 18–64. The legislative framework, notably the Genetic Information Nondiscrimination Act (GINA), provides relatively limited protection, including health insurance and job discrimination, but leaves out long‐term care, life, and disability insurance coverage [33]. These issues are magnified on a global scale, where developed nations' emphasis on diseases affecting their populations, combined with their greater financial resources for sophisticated medical interventions, perpetuates a cycle in which genetic research primarily benefits already‐privileged populations, exacerbating existing healthcare disparities [34, 35].

### 3.3 Gender Disparities as a Challenge for Precision Medicine

The development of precision medicine must actively address and prevent the perpetuation of gender gaps in healthcare, as historical evidence demonstrates the harmful consequences of gender‐blind medical research [36]. A striking example from the 1990s shows that 80% of drugs withdrawn from the US market had disproportionately negative effects on women, highlighting the potentially disastrous outcomes when gender‐specific factors are overlooked [37]. Currently, precision medicine faces several critical gender‐specific challenges that require attention. First, research design must ensure accurate representation of gender and sex differences in clinical trials and data collection. Second, women's health needs special consideration in areas such as pregnancy, postpartum care, chronic disease management, and reproductive health [38]. Third, existing social and economic barriers that limit women's access to medical treatment may also restrict their access to precision medicine. Fourth, healthcare resources must be developed to address the distinct biological requirements of both men and women. This is particularly evident in the current gaps in managing conditions like pregnancy‐related hypertension [39]. While these gender disparities in healthcare are universal, they are notably more severe in regions where women face additional barriers to medical access [25]. The ultimate challenge lies in developing and implementing precision medicine in a way that advances women's health and reduces healthcare gender gaps, requiring careful consideration of both biological sex differences and sociocultural gender norms [40].

### 3.4 The Digital Divide

Advanced technologies, including artificial intelligence, proteomics, and genomes, have been tailored by precision medicine to improve diagnosis, treatments, and preventative measures [41]. Implementing new technologies brings about challenges since users might find it difficult to fully incorporate them into their workflows [40]. One clear example was the failed £12 billion effort to develop a nationally integrated electronic patient record system in 2010 by the UK Department of Health, which revealed enormous infrastructure difficulties in the deployment of precision medicine [41]. Physicians find it hard to incorporate information technology (IT) systems into their work practices, which causes them to either not use the system at all or only employ parts that they find useful [40]. A number of serious issues with creating precision medicine information systems are brought to light by this setback. On a more technical level, healthcare providers have challenges when trying to create efficient platforms for health data management and when attempting to integrate and filter massive data sets from many sources [4]. In intensive care units, where time and precision are of the essence, the lack of computerized decision support systems (CDSS) slows down clinical implementation [42]. The absence of standards based on evidence, privacy concerns, and data governance challenges makes it especially difficult to integrate these technologies at the point of care [43]. Integrating patient genotyping data into electronic health records (EHR), communicating effectively with patients and decision‐makers, and providing fast and accurate diagnoses are all complicated demands of precision medicine that the system must meet. Factors unique to each condition and the preferences of individual patients seeking therapeutic counseling add another layer of complexity to this integration process. Even massive, well‐funded efforts to establish precision medicine infrastructure can be derailed by these complex issues, as seen in the UK case [41, 44].

### 3.5 Education and Awareness

Precision medicine education and awareness are essential for the public and healthcare professionals. Clinical practice may be less accessible due to misperceptions, mistrust, and unwillingness to adopt new technologies. Lack of doctor pharmacogenomics knowledge hinders implementation. Despite advancements, the public's understanding remains inadequate. University of Nevada survey reveals college students like precision medicine (PM) but are unfamiliar with it, highlighting the need for genetic testing in the future healthcare workforce and stakeholders [45]. The Genomic Medicine Institute at the Cleveland Clinic and Mayo Clinic's Center for Individualized Medicine are educating clinicians, but their programs have not yet reached many providers [31]. Regarding the general population, especially underrepresented populations such as those living in rural areas, there is a genomic health illiteracy. Though neglected, genomic health literacy affects PM engagement, from research to risk assessments and medical advice to policy discussions regarding data use and PM in healthcare [46]. Genomic health literacy is described as the ability to acquire, interpret, comprehend, and utilize genomic information for health‐related decision‐making [46]. When genomic risk information is conveyed in a manner that exceeds individuals' comprehension skills or linguistic capabilities, it is improbable to influence behavior effectively; alternatively, it may alter behavior in unanticipated ways without ultimately enhancing health or environmental consequences [47]. Health literacy influenced precision medicine knowledge and attitudes more than race and ethnicity [48, 49]. While implementing precision medicine education in rural areas, education program materials must be culturally suitable and audience‐specific to ensure cultural competence. This includes overcoming cultural and linguistic gaps, and rural health promotion and disease prevention hurdles [50].

### 3.6 Privacy and Informed Consent Concerns

Concerns about privacy and informed consent in EHR systems have also emerged as a problem for precision medicine. Data breaches in these platforms potentially reveal sensitive personal and health information [50]. Specific informed consent concerns include the medical field's limited ability to clinically address genomic testing results, identifying appropriate detail levels in consent processes [51], introducing new consent formats, and managing patients' expectations regarding genomic testing [52]. Furthermore, legal constraints involving information accessibility, notably insurability, provide additional impediments to adoption [13]. In addition to the permission issue, easy access to the genetic information of people by third parties such as insurers raises questions regarding discrimination. The findings of genetic tests may be used as grounds for discrimination by health and life insurance companies. With the use of this data, insurers may be able to identify with remarkable accuracy which people are most likely to have longer lifespans or spend the highest medical expenses. As a result of this, they may charge higher rates or refuse coverage to people who are thought to be high‐risk [53]. Variable coverage policies and different testing criteria across nations add an additional layer of complication to the insurance and policy structure [54]. As of right now, 47 nations have enacted antidiscrimination insurance plans based on genetic information [55]. These policies take quite different approaches; for example, the GNA in Canada covers both diagnostic and predictive testing [27], while the Code on Genetic Testing in the UK is purely concerned with predictive testing [56].

### 3.7 Challenges for Regulatory Frameworks

PMI's framework offers a well‐defined strategy for implementation and strong financing, complementing the numerous large‐scale precision medicine application development initiatives spurred by the HGP and genetic advancements. The PMI Cohort Program was announced by NIH Director Francis Collins in September 2015 [57].

One million people are being sought by the All of Us Research Program to gather data from clinical trials, EHR, DNA samples, and blood samples for genetic analysis [58]. The Federal Drug Administration has issued a comprehensive set of rules that control the whole cycle of pharmaceuticals and biological products. We need separate but complementary shorter routes to market for pharmaceuticals (drugs) and biologics (biotech) due to the complexity of biological goods and the established legislative frameworks [59].

Over the next decade, the National Institutes of Health (NIH) will receive $4.8 billion in funding for the PMI. This substantial boost in federal funding for precision medicine was made possible in December 2016 by the 21st Century Cures Act. With allocated money, the PMI is established under the 21st Century Cures Act, which also authorizes the HHS secretary to begin the program and promote innovation in healthcare [60].

Some laws have made minor but substantial changes to the statutory framework of the FDCA and PHSA; for example, the Biologics Price Competition and Innovation Act of 2009 (which is a part of the Affordable Care Act) and the Food and Drug Administration Safety and Innovation Act of 2012, among others. Some examples of these changes include the recognition of specific drugs as breakthrough medicines and the commercialization of biosimilar and interchangeable biologic pharmaceuticals. The FDA is implementing the framework for regulations in light of these changes in legislation [60].

An increasing number of precision medicine medications have been approved by the FDA, demonstrating their dedication to utilizing molecular data to personalize patient treatments [61].

The Food and Drug Administration (FDA) uses its three medical product review centers—CBER, CDRH, and CDER—to keep precision medicine items under review. Many of the statutory authorities that each center uses to enforce laws have been in place for quite some time. The intricacies of personalized medicine, however, are not well addressed by these rules. Various products depend on one another for safety and effectiveness. Laws pertaining to precision medicine products are so inconsistent [61].

Regulatory frameworks need to change to accommodate the intricacies of diagnostic tools and medicines that involve multiple components as personalized medicine progresses. To promote innovation while guaranteeing the efficacy and safety of individualized medicines, regulatory agencies such as the FDA will have to create more integrated and adaptable standards. Data privacy, fair access to individualized treatments, and informed consent are all ethical concerns that will grow in significance. In order to ensure that patients' rights are protected and that tailored healthcare is provided fairly, lawmakers and healthcare providers must collaborate to create strong protections [61, 62].

## 4 Strategies for Eliminating Inequities and Expanding Access to Precision Medicine

Investing in education, promoting genomic data diversity, expanding access to precision medicine for underprivileged groups, and establishing regulatory frameworks can help deliver better health outcomes in precision medicine [63]. Before providing target drugs, the healthcare system should check patients for pharmacogenetic variation. Variant DNA data would be added to the patient's EHR and linked to clinical decision‐making tools to guide medication prescribing. Regular clinical care creates organized and unstructured EHR data. Linkage and integration of varied data can create the “human phenome sequence”—a fine‐grained longitudinal picture of health throughout time. Data diversity may improve clinically relevant disease, etiology, and classifier cluster resolution. Big data research requires searchable data catalogs, metadata, feasibility counts (and sample data), and access agreements [64]. The complexity of such an approach is evident. Expert design of the test, expert curation of projected implications of the genetic variants, clinical knowledge in drug prescribing and alternatives, and technical experience to support laboratory testing, reporting, and decision support; all of these are necessary for successful pharmacogenomic evidence‐based medicine implementation.

To enhance health outcomes for all segments of the population, genomic applications must be implemented with strong public health leadership to lay the groundwork with evidence and provide direction. Communities impacted by social determinants of health, such as racism, must have their specific difficulties evaluated [65]. Policy hurdles to the efficient, broad deployment and oversight of genomic applications necessitate public health measures as well.

The necessity for a health equity agenda in genomics research is crucial to guarantee that genomic discoveries represent all population. Integrating genomic techniques into community and healthcare settings helps public health address genetic health inequalities. By engaging communities fairly and resolving genomic medicine implementation inequities, public health programs and health systems can enhance population health outcomes [66].

The infrastructure for precision medicine still needs to be constructed, but the resources that have been invested until now will allow people to get the health and economic benefits of matching each patient with the proper or preventive therapy [67]. Mobile technology, including wearable and environmental sensors, may also help collect key data that could be used in a “learning health care system,” a seamless system that would capture, analyze, and share findings from every clinical interaction and research milestone. Patients require genetic data privacy and education on personalized medicines. This data must be collected and communicated using best practices to ensure clinical therapy. The fee‐for‐service paradigm may need to be adjusted to accommodate new technology and ensure patient access to care, and payers require education to understand the evidence needed to make favorable coverage decisions [34]. Moreover, using artificial intelligence, machine and deep learning approaches, and neural networks is one of the solutions to overcome challenges and barriers in personalized medicine [38]. Automated estimations could potentially help evaluate remission or illness progression over time and increase our understanding of therapy efficacy in real life. For example, Alves presented a machine‐learning algorithm that used clinical data to generate four SLEDAI score categories for systemic lupus erythematosus (SLE) patients. The model's output was connected with the prescription of steroids and analgesics as well as the utilization of medical resources [68]. Precision medicine needs broad engagement, trust, privacy protection, and value return for huge populations. Participants must trust data privacy and security. Laws and technologies like de‐identification, blockchain, hashed identifiers, and homomorphic encryption help secure information systems [13].

To address gender disparities in personalized medicine, researchers should ensure inclusive representation, conduct gender‐specific analysis, and receive education. Using methods aligned with precision medicine goals, such as a “sex contextualist” approach, can improve the study of sex‐related factors, recognizing their diversity and context‐specificity [69].

Wearable devices can enhance antenatal care by improving maternal and newborn health, but studies often focus on a small number of pregnant women without complications. Pregnancy‐related pathophysiological responses vary, requiring deeper analysis of medical history. AI implementation in multimodal assessment could enhance pregnancy‐related disorders and personalized treatment planning, utilizing large patient data sets to provide preventive advice [27]. Wearable technology can extend life years that are quality‐adjusted while also being reasonably priced and possibly even cost‐saving [70].

Here is an example of the incorporation of precision medicine and overcoming the challenges of its application. WWE and their families should undergo genetic counseling to comprehend the predictive value, clinical utility, limitations, and ethical and legal implications of genetic testing. According to the “Nothing about us, without us” tenet, it is only fair that pregnant women, who will be directly affected by the results of personalized medicine, have a hand in its development and distribution [31].

## 5 Discussion

The integration of precision medicine into clinical practice holds transformative potential, yet its inequitable implementation perpetuates disparities, particularly for vulnerable populations such as WWE. The case of WWE underscores the intersection of gender‐specific challenges, ethical considerations, and systemic barriers that hinder access to personalized care. As highlighted in this manuscript, WWE face unique risks during pregnancy, where ASMs may pose teratogenic effects, yet pharmacogenetic insights remain understudied for most ASMs [22]. This gap exemplifies broader issues in precision medicine: underrepresented populations are often excluded from genetic research, limiting the applicability of findings and exacerbating health inequities [29, 30]. For instance, while genetic counseling could empower WWE to make informed decisions about ASM use, disparities in genomic literacy and access to testing—particularly among low‐income or minority groups—restrict this opportunity [22, 46].

The “Nothing about us, without us” principle [56] emphasizes the necessity of involving patients like WWE in designing precision medicine interventions. Their lived experiences can guide the development of culturally sensitive, accessible tools for genetic risk assessment and antenatal care. However, this requires addressing structural barriers such as the digital divide [41] and socioeconomic constraints [24], which disproportionately affect marginalized communities. For example, wearable technologies and AI‐driven monitoring systems [27, 67] could enhance personalized antenatal care for WWE, yet these innovations remain inaccessible in resource‐limited settings.

Furthermore, the ethical and regulatory challenges outlined in this manuscript—such as genetic discrimination by insurers [54] and fragmented regulatory frameworks [60]—are magnified in populations like WWE. Robust policies are needed to ensure genetic data privacy while promoting equitable access to targeted therapies. The GINA offers limited protections [33], underscoring the urgency of global reforms to safeguard against misuse of genomic information.

Strategies proposed in the conclusion, including education, genomic data diversity, and regulatory harmonization, are critical to bridging these gaps. For WWE, tailored educational programs could improve pharmacogenomic literacy among patients and providers, enabling shared decision‐making [22, 46]. Meanwhile, diversifying genomic databases to include underrepresented populations [26, 65] would enhance the validity of ASM safety profiles across diverse genetic backgrounds. Regulatory agencies must also expedite the integration of precision medicine into standard care pathways, ensuring therapies like GLP‐1 analogs [16] or immune‐targeted interventions [20] are accessible to all.

In summary, the case of WWE illustrates how precision medicine's promise is tempered by systemic inequities. Addressing these disparities requires a multifaceted approach: empowering patients through education, prioritizing inclusive research, dismantling structural barriers to access, and enacting equitable policies. By centering the needs of vulnerable populations in PMIs, healthcare systems can move closer to achieving the paradigm of “right treatment, right patient, right time” for all [1, 66].

## 6 Conclusion

Precision medicine has successfully treated and helped early detection of many diseases like asthma, cancer, and type 1 diabetes. Minority and low‐income communities cannot access it due to disparities in socioeconomic status, education, data, access, and regulatory frameworks. To fully benefit all, investment in education, genetic data diversity promotion, and increased access, the patient‐oriented strategy is cost‐effective and includes benefits through education, genetic data diversity promotion, and increased access, but regulatory frameworks are essential.

## Author Contributions

**Maha Hosni Morsi:** conceptualization, validation, writing – review and editing. **Bashaer Elawfi:** writing – original draft, writing – review and editing. **Saad Ashraf ALsaad:** writing – original draft. **Ahmed Nazar:** writing – original draft. **Hamed Abdelma'aboud Mostafa:** writing – original draft. **Sara Adel Awwad:** writing – original draft. **Maya Magdy Abdelwahab:** writing – original draft. **Husam Tarakhan:** writing – original draft. **Ehssan Baghagho:** writing – review and editing. The final manuscript is a collaborative effort of all contributors, which helped shape the research. All authors have approved the final draft.

## Conflicts of Interest

The authors declare no conflicts of interest.

## Transparency Statement

The lead author, Maha Hosni Morsi, affirms that this manuscript is an honest, accurate, and transparent account of the study being reported; that no important aspects of the study have been omitted, and that any discrepancies from the study as planned (and, if relevant, registered) have been explained.

## Data Availability

The authors have nothing to report.
